# Editorial: Calcium: An Overview From Physiology to Pathological Mineralization

**DOI:** 10.3389/fendo.2022.932019

**Published:** 2022-06-24

**Authors:** Volha V. Zhukouskaya, Claire Bardet

**Affiliations:** ^1^ Université Paris Cité, Laboratory of Orofacial Pathologies, Imaging and Biotherapies URP2496 and FHU-DDS-Net, Dental School, and Plateforme d’Imagerie du Vivant (PIV), Montrouge, France; ^2^ Assistance Publique Hôpitaux de Paris (APHP), Department of Diabetology and Clinical Immunology, Hôpital Cochin, Paris, France

**Keywords:** calcium, vitamin D, parathyroid hormone, kidney, dental fluorosis, denosumab, ectopic mineralization, enamel renal syndrome

Widely involved in cell signalling, either by direct signal transduction or acting as a second-messenger, calcium regulates a large range of physiological cell functions and processes including regulatory effects on many enzymes and proteins, muscle contraction, neuronal transmission and genesis, cellular motility and growth. Calcium, together with phosphate, also participates in the mineralization of calcified tissues where bones act as calcium storage site, intimately linked with the preservation of calcium balance within the non-bone tissues of the body.

In order to maintain calcium at a constant level, cells have developed a complex machinery. The regulation of calcium homeostasis, presented on the **Figure 1**, involves three major organs and several hormones, namely the parathyroid hormone (PTH), secreted by the parathyroid glands, and the active form of the vitamin D (1,25-dihydroxyvitaminD_3_), produced by the proximal tubular cells in the kidney. The regulation of PTH secretion in response to serum calcium variation is monitored by the calcium sensing receptor (CaSR). Although PTH secretion and action has been long known, the activity of the CaSR was characterized recently. Additionally, the role of FGF23, a molecule associated with phosphate metabolism, remains limited in calcium homeostasis (1).

**Figure 1:**
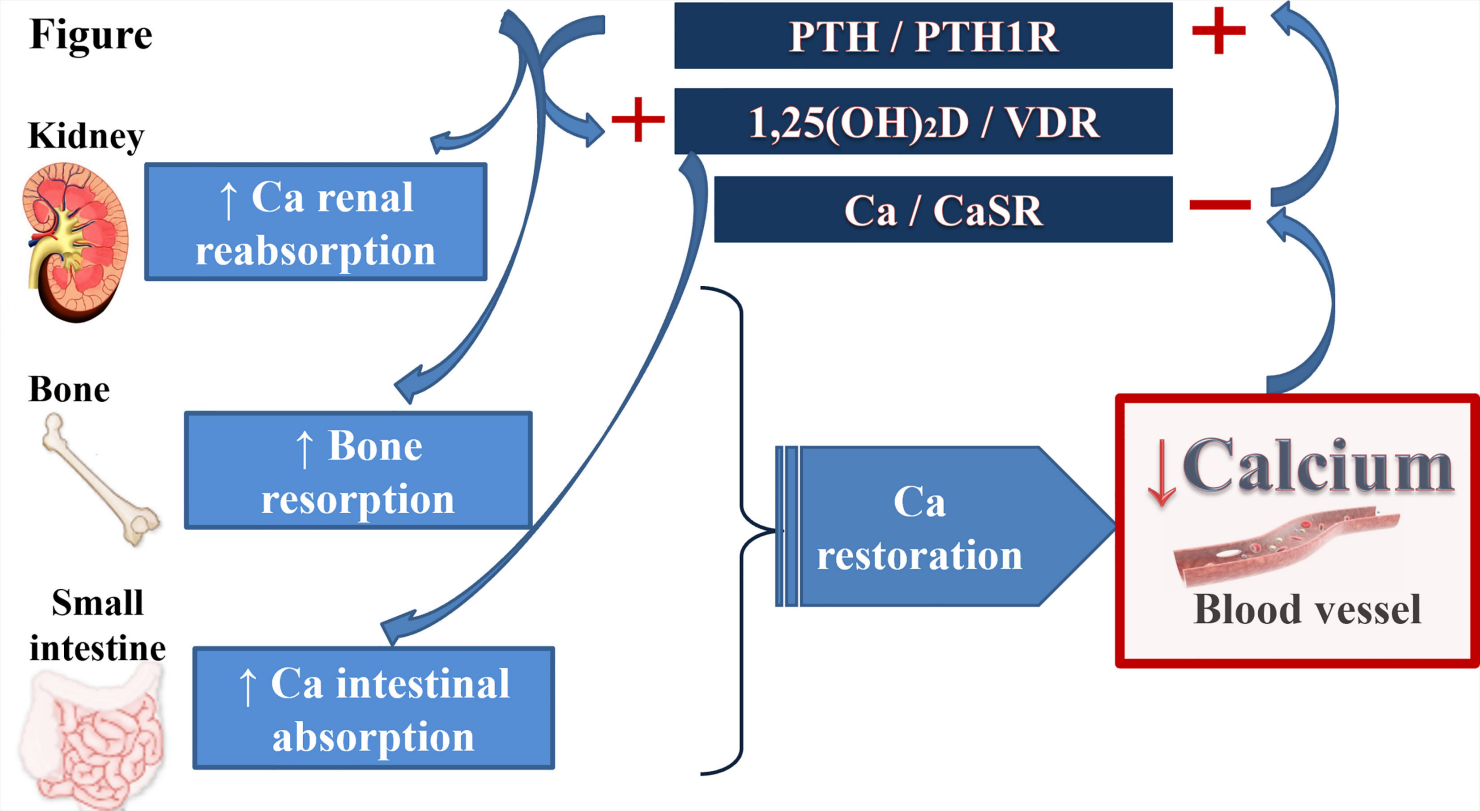
Calcium homeostasis. Calcium homeostasis is finely regulated by a complex hormonal system including several hormones and its receptors (PTH/PTHrP and its receptor PTH1R; 1,25(OH)_2_D) and the vitamin D receptor; ionized calcium itself and CaSR) and 3 main target-organs (kidney, gut and bone). A decrease in serum calcium inactivates the CaSR in the parathyroid glands and in the kidney. It causes an increase in PTH secretion which in turn, stimulates renal and bone reabsorption. PTH promotes the synthesis of 1,25(OH)_2_D in the kidney leading to the increase of intestinal calcium absorption and to a feedback loop inhibition of PTH secretion. Inactivation of CaSR in the kidney leads to additional calcium reabsorption and enhancement of the renal PTH action of PTH. In contrast, an increase in serum calcium level increases calcium excretion and bone storage. PTH, parathyroid hormone; PTHrP, PTH-related Protein; PTH1R, PTH-1 receptor; CaSR, calcium-sensing receptor; 1,25(OH)_2_D, 1,25-dihydroxyvitamin D; VDR, vitamin D receptor.

Despite recent advances, the regulation of calcium homeostasis remains to be addressed in either physiological or pathological conditions. Here we present an exciting journey into what has been recently done in this field.

In the review *“Calcium transport in the kidney and disease processes”*, Hanna et al.  discussed about the importance of the kidney in the whole-body calcium balance, and how drugs, hormone dysregulation and genetic mutations in key proteins from different nephron segments resulted in various diseases processes.

Continuing in the same direction, Molin et al. presented an original article, *“Overlapping phenotypes associated with CYP24A1, SLC34A1 and SLC34A3 mutations: a cohort study of patients with hypersensitivity to vitamin D*” with extensive clinical, biochemical and molecular data on a large French cohort of patients with the vitamin D hypersensitivity, namely Infantile Hypercalcemia type 1 and 2. Mutations in *CYP24A1* (vitamin D 24-hydroxylase) and *SLC34A1* (renal phosphate transporter NPT2a) have been recognized as the main causes of this clinical entity. The authors have explored the contribution of other genes, normally involved in the renal phosphate transport, in the clinical manifestation of this rare condition, thus providing the knowledge about intriguing complexity of calcium regulation in the kidney.

Recent research has focused on understanding the intracellular, transcellular and paracellular pathways of calcium transport. Indeed, several mutations in some of the components of intracellular calcium influx (e.g., *ORAI1, STIM1*) are associated with distinct disease syndromes, channelopathy including amelogenesis imperfecta, immune dysfunction, ectodermal dysplasia, muscle weakness and anhidrosis (2). *In the review “Calcium transport in specialized dental epithelia and its modulation by fluoride”*, Costiniti et al. discovered the most relevant aspects of what is known about calcium transport in dental enamel forming cells (the ameloblasts), focusing on the effects of a worldwide phenomenon caused by excessive amounts of fluoride, and how it can alter the calcium function leading to dental fluorosis.

Several drugs commonly used in clinical practice such as diuretics, proton pump-inhibitors, glucocorticoids, lithium, bisphosphonates, high doses of vitamin A and D, are also known to alter calcium homeostasis causing hyper- or hypocalcaemia. Denosumab, a fully monoclonal antibody against RANKL (Receptor Activator Nuclear Factor-kB Ligand) widely used for post-menopausal osteoporosis through suppression of bone resorption, has demonstrated its efficacy in other rare pathological entities as giant cell tumours, aneurysmatic bone cysts and several other osteolytic diseases (3, 4, 5). Beyond the hypocalcaemia frequently seen in adults upon the denosumab, rebound hypercalcemia at discontinuation of this treatment may also happen in children. Del Sindaco et al. *illustrated a child case report on mineral and bone consequences of high dose denosumab to treat an aneurysmal bone cyst*.

Finally, little is known about a regulatory system preventing the harmful deposition of ectopic calcium phosphate complexes in soft non-mineralized tissues. The Enamel Renal Syndrome is a rare disorder due loss-of-function mutation in the *FAM20A* gene which is characterized by ectopic accumulation of calcium phosphate complexes within gingival, pulp and renal tissues (6). The new pathogenetic pathways implicated in this rare condition has been revealed by Escorcia et al. *in the original article “Pathogenesis of enamel-renal syndrome associated gingival fibromatosis: a proteomic approach*”  in particular how proteins of the extra-cellular matrix influence the ectopic calcifications.

## Author Contributions

VVZ designed and prepared the manuscript, CB designed and reviewed the mansucript. All authors contributed to the article and approved the submitted version.

## Conflict of Interest

The authors declare the absence of any commercial or financial relationships that could be construed as a potential conflict of interest.

## Publisher’s Note

All claims expressed in this article are solely those of the authors and do not necessarily represent those of their affiliated organizations, or those of the publisher, the editors and the reviewers. Any product that may be evaluated in this article, or claim that may be made by its manufacturer, is not guaranteed or endorsed by the publisher.
